# The Association between Glymphatic System and Perivascular Macrophages in Brain Waste Clearance

**DOI:** 10.3390/diagnostics14070731

**Published:** 2024-03-29

**Authors:** Jasleen Kaur, Edward D. Boyd, Guangliang Ding, Li Zhang, Hao Luo, Qingjiang Li, Lian Li, Min Wei, Julie Landschoot-Ward, Michael Chopp, Zhenggang Zhang, Quan Jiang

**Affiliations:** 1 Department of Neurology, Henry Ford Health System, Detroit, MI 48202, USA; jkaur3@hfhs.org (J.K.); eboyd3@hfhs.org (E.D.B.); gding1@hfhs.org (G.D.); lzhang3@hfhs.org (L.Z.); hluo2@hfhs.org (H.L.); qli1@hfhs.org (Q.L.); lli2@hfhs.org (L.L.); mwei1@hfhs.org (M.W.); jlandsc1@hfhs.org (J.L.-W.); mchopp1@hfhs.org (M.C.); zzhang1@hfhs.org (Z.Z.); 2 Department of Physics, Oakland University, Rochester, MI 48309, USA; 3 Department of Radiology, Michigan State University, East Lansing, MI 48824, USA; 4 Department of Physiology, Michigan State University, East Lansing, MI 48824, USA; 5 Department of Neurology, Wayne State University, Detroit, MI 48202, USA

**Keywords:** glymphatic system, perivascular macrophages, MRI, T1-weighted imaging, diffusion tensor imaging, confocal microscopy imaging

## Abstract

The glymphatic system suggests the convective bulk flow of cerebrospinal fluid (CSF) through perivascular spaces and the interstitial spaces of the brain parenchyma for the rapid removal of toxic waste solutes from the brain. However, the presence of convective bulk flow within the brain interstitial spaces is still under debate. We first addressed this argument to determine the involvement of the glymphatic system in brain waste clearance utilizing contrast-enhanced 3D T1-weighted imaging (T1WI), diffusion tensor imaging (DTI), and confocal microscopy imaging. Furthermore, perivascular macrophages (PVMs), which are immune cells located within perivascular spaces, have not been thoroughly explored for their association with the glymphatic system. Therefore, we investigated tracer uptake by PVMs in the perivascular spaces of both the arteries/arterioles and veins/venules and the potential association of PVMs in assisting the glymphatic system for interstitial waste clearance. Our findings demonstrated that both convective bulk flow and diffusion are responsible for the clearance of interstitial waste solutes from the brain parenchyma. Furthermore, our results suggested that PVMs may play an important function in glymphatic system-mediated interstitial waste clearance. The glymphatic system and PVMs could be targeted to enhance interstitial waste clearance in patients with waste-associated neurological conditions and aging.

## 1. Introduction

The glymphatic system is a newly discovered brain-wide waste clearance pathway which suggests that cerebrospinal fluid (CSF) from subarachnoid space enters the brain parenchyma along the penetrating arteries, mixes with interstitial fluid (ISF) and interstitial waste solutes, and drains along the veins through a size-independent convective bulk flow within the perivascular spaces [1,2,3,4,5,6,7,8]. Aquaporin-4 (AQP4) protein water channels located at the astrocyte end-feet promote the convective bulk flow of CSF in the interstitial spaces between the periarterial and perivenous routes [1,9]. Cerebral arterial pulsations are thought to generate pressure variations, which also contribute to CSF convective bulk flow and promote CSF-ISF exchange in the glymphatic system [10,11]. Vasomotions, which are low-frequency (~0.1 Hz) spontaneous vasodilations and vasoconstrictions produced by vascular smooth muscle cells, independent of pulsatile blood flow are also considered a driving force for glymphatic CSF flow [12]. Additionally, parenchymal border macrophages (PBMs) (including the perivascular macrophages (PVMs)) and leptomeningeal macrophages) regulate the CSF flow via extracellular matrix (ECM) remodeling, which influences arterial stiffness and pulsations [13]. Also, the dynamic changes in arterial diameter due to functional hyperemia/neurovascular coupling and arterial vasomotions accelerate the glymphatic CSF influx and clearance [14]. 

Similar to the glymphatic hypothesis, some experiments in the past demonstrated the convective bulk flow of solutes, but it was limited to the perivascular spaces rather than the whole interstitial space [15,16,17,18,19]. The convective bulk flow of tracers was also observed along the white matter fiber tracts [15,20,21,22,23]. More recent research [24] and modeling techniques demonstrate a size-dependent diffusive transport of solutes throughout the brain parenchyma instead of convective bulk flow [25,26]. Similarly, multiple previous investigations using various molecular-weight tracers have established that diffusion gradients drive solute transport from the brain parenchyma to nearby CSF spaces [23,27,28,29,30,31,32]. However, it is questionable if the convective bulk flow is limited to the perivascular spaces or occurs within the brain interstitial space alongside diffusion.

The glymphatic system utilizes the perivascular spaces for CSF-ISF exchange and clearance of interstitial waste solutes from the brain parenchyma. PVMs are a specific subpopulation of myeloid cells which are located in the perivascular spaces between the vascular basement membrane and glia limitans of blood vessels. They are constantly in direct contact with the CSF and play a significant role in brain homeostasis [33,34,35,36,37,38]. PVMs were long thought to be produced from bone marrow precursor cells that enter the brain via the bloodstream and are continually replaced by blood monocytes [39]. Recent research, however, indicates that PVMs are formed early during development by embryonic precursors derived from the yolk sac and are not replaced by blood monocytes [38,40]. It is important to keep in mind that, despite substantial studies on the glymphatic system and PVMs, these topics are an area of ongoing investigation for better understanding.

Both the glymphatic system and the PVMs play important functions in a variety of neurological conditions. The glymphatic system has been correlated with numerous neurological conditions such as Alzheimer’s disease (AD) [41,42,43], traumatic brain injury (TBI) [5,44,45,46,47,48,49], microinfarcts [50,51], small vessel disease (SVD) [52,53], migraine [54], stroke [55,56,57], diabetes [58], glaucoma [59]. Moreover, aging [60,61], sleep [45], anesthesia [62], body posture [63], etc. influence the glymphatic system function. On the other hand, PVMs are scavenger/immune cells that help preserve blood-brain barrier (BBB) integrity [64], participate in macromolecule/pathogens/cellular debris uptake (phagocytosis) [65,66,67,68], and antigen presentation to lymphocytes [39,69,70]. They constitute a vital component of the brain’s immune system [33,71,72,73] and play a role in a range of neurological conditions such as AD [74,75,76], ischemic stroke [77,78], multiple sclerosis [79,80,81], brain infections [82,83,84,85,86], hypertension [87,88], etc. Recent promising research has demonstrated the critical function of PBMs in AD and aging; PBMs depletion in mice leads to a considerable buildup of amyloid-beta (Aβ) plaque (an AD-related protein); in aged-mice macrophage colony-stimulating factor (M-CSF) treatment significantly increases MMP activity and reduces the deposition of extracellular matrix (ECM) protein [13]. 

Utilizing in-vivo T1-weighted imaging (T1WI) with Gd-albumin contrast, diffusion tensor imaging (DTI), and confocal microscopy imaging, the objectives of this study are to determine (1) if diffusion is sufficient to account for the intra-striatally administered tracer dynamics within the brain interstitial spaces, particularly, the striatum and the corpus callosum of the rat brain, (2) the role of the glymphatic system in intra-parenchymal tracer transport, (3) tracer uptake by PVMs along both the arteries and veins and, (4) the association between the glymphatic system and the PVMs.

## 2. Materials and Methods

All experimental procedures were conducted and performed in accordance with guidelines of the National Institute of Health (NIH) for animal research under a protocol approved by the Institutional Animal Care and Use Committee of Henry Ford Hospital, and experimental guidelines of ARRIVE (items 8, 10 to 13).

### 2.1. Animals and Surgical Procedures

#### 2.1.1. Rats

Wistar Rats (3 months, male, Charles River, Wilmington, MA, USA) were subjected to MRI measurements and ex-vivo fluorescent tracer distribution study using confocal microscopy after intra-striatal tracer infusion (*n* = 10) and intra-cisternal tracer infusion (*n* = 3).

#### 2.1.2. Intra-Striatal Surgical Procedure

Anesthesia was induced with 3.0% Isoflurane in a gas mixture of N_2_O (70%) and O_2_ (30%) and maintained with 1.0–1.5% Isoflurane throughout the procedure. For intra-striatal fluorescent tracer (FITC-Dextran, 70 kD) and MRI contrast agent (Gd-albumin, 70 kD) infusion, anesthetized rats were placed in a stereotaxic frame (Stoelting Co., Wood Dale, IL, USA) and the skin was opened to expose the skull. A small burr hole was made over the right parietal cortex with a hand-held drill (Foredom Electric Co., Bethel, CT, USA). A 27-gauge needle was introduced into the right striatum at the following coordinates based on the rat brain atlas (Paxinos and Watson, 1997): anterior-posterior (AP) = 0 mm; midline (ML) = 3.5 mm; vertical depth (VD) = 5 mm from bregma. FITC-Dextran (3 μL constituted in artificial CSF at a concentration of 1%) and MRI contrast agent, Gd-albumin (3 μL) were loaded together into a PE-10 catheter (Becton Dickinson, MD, USA) and part of the catheter was introduced through the needle track into the striatum. The skull burr hole was then sealed with bone wax. Rats were taken to the 7 Tesla (T) magnet for MR imaging immediately after the surgical procedure. The remaining part of the indwelling catheter was connected to another PE-10 catheter filled with saline and attached to a glass Hamilton syringe as an extension out of the MRI machine.

#### 2.1.3. Intra-Cisternal Surgical Procedure and Tracer Infusion

Anesthetized rats were placed in a stereotaxic frame (Stoelting Co.) and the atlanto-occipital regions covering the cisterna magna were exposed by tilting the head downward at ~90° angle. The occipital bone was exposed via a midline incision. A small burr hole was made over the occipital bone 1 mm above the cisterna magna and 1 mm lateral to the midline. The dura mater was carefully exposed and punctured with a 27-gauge needle. A polyethylene catheter (PE-10 tubing; Becton Dickinson, MD, USA) filled with 30 μL of fluorescent tracer (FITC-dextran, 70 kD) was introduced into the cisterna magna through the puncture to a depth of 1–2 mm with its tip positioned at the midline of cisterna magna. The indwelling catheter was secured, and the perforation site was sealed with surgical glue. The skin incision was then stitched. Using the indwelling catheter connected to a Hamilton syringe, FITC-dextran was administered at a rate of 1 μL/min for 30 min using an infusion pump (Harvard Apparatus, Holliston, MA, USA). To avoid possible backflow, the indwelling catheter and Hamilton syringe were left in place after infusion. Rats were sacrificed 2 h after infusion to study the dynamics of FITC-dextran in the coronal brain sections using confocal microscopy imaging.

### 2.2. MRI Measurements

A 7 Tesla MRI system (Bruker-Biospin, Billerica, MA, USA) was employed to perform MRI measurements. The receiver was a quadrature 2 × 2 surface array coil, while the transmitter was a body volume birdcage-type coil. Rats were safely placed on an MR-compatible holder equipped with stereotaxic ear bars to control head motions and an adjustable nose cone for anesthetic gas distribution. At the start of the MRI scan, the holder was inserted into the magnet and placed in the middle. Isoflurane (1.0–1.5%) and a gas mixture of N_2_O (70%) and O_2_ (30%) were utilized as anesthesia during the MRI measurements, and respiration (50–65 breaths/minute) was carefully observed throughout the MRI scanning (Biopac Systems Inc., Goleta, CA, USA). The rectal temperatures of the rats were maintained at 36 °C with an air heating blower (Rapid Electric, Brewster, NY, USA).

To measure the dynamics of the Gd-albumin after intra-striatal infusion, 3D T1WI (TE = 4 ms, TR = 18 ms, flip angle = 12°, FOV = 32 × 32 × 16 mm^3^, matrix = 256 × 192 × 96 (resolution of 0.125 × 0.167 × 0.167 mm) later interpolated to 256 × 256 × 96 voxels (resolution of 0.125 × 0.125 × 0.167 mm)) were acquired using a spin-echo sequence. Two baseline scans using 3D T1WI sequences were employed prior to infusion, followed by intra-striatal administration of Gd-albumin + FITC-dextran (6 μL, 70 kD) at an infusion rate of 0.3 μL/min for 20 min using the indwelling catheter connected to a 100 μL glass syringe (Hamilton Robotics, Reno, NV, USA) mounted on an infusion pump (Harvard Apparatus, Holliston, MA, USA). After the end of an infusion, three 3D T1WI sequences were employed to track the movement of Gd-albumin. Diffusion-weighted (DW) spin-echo sequence to estimate ADC of water molecules was also employed with parameters TE = 40 ms, TR = 1500 ms, b-value = 900 s/mm^2^, directions = 6, slice thickness = 0.8 mm, number of slices = 15, FOV = 32 × 32 mm^2^, matrix = 128 × 128. To avoid possible backflow, the Hamilton syringe was left in place after infusion. At the end of the experiment, animals were immediately taken for sacrifice, and tissues were prepared for ex-vivo confocal microscopy imaging. All rats were anesthetized intraperitoneally with ketamine/xylazine prior to sacrifice. Anesthesia was verified by the absence of a righting reflex and a reaction to toe pinching. Rats were then transcardially perfused with saline, followed by 4% paraformaldehyde. The rats were sacrificed by decapitation using a standard small-animal guillotine device and brain tissues were harvested.

### 2.3. Histology

#### 2.3.1. Tissue Preparation for Vibratome Sections and Immunohistochemistry

The brains were immersed in 4% paraformaldehyde at 4 °C for 48 h. Coronal vibratome sections (100 μm, AP = 0.20 to −0.4 mm from bregma) for rats with intra-striatal infusion and intra-cisternal infusion were processed for single immunofluorescence labeling for CD206 and alpha-smooth muscle actin (αSMA). Briefly, vibratome coronal brain sections were incubated with the primary antibody including CD206 (PVMs marker, Abcam, AB64693, 1:2000) or αSMA (smooth muscle cells marker, DAKO, M0851, 1:400) for 4 days at 4 °C, and then with the secondary antibody conjugated to Cy3.

#### 2.3.2. Ex-Vivo Confocal Microscopy Imaging

Coronal brain sections were assessed using an Olympus FLUOVIEW FV1200 Biological Confocal Laser Scanning Microscope (Olympus America Inc., Center Valley, PA, USA). The FV10-ASW software (Version: 4.1a) was used to operate the motorized stage, focus, and XYZ image acquisition. The distribution of FITC-dextran was imaged with FITC (green) emission channel and CD206 or αSMA staining samples were imaged using cy3 (red) emission channel. We used Olympus UPlanSApo 10×/0.40 and 40×/0.95 objectives and laser lines at the excitation wavelength of 473 nm and emission wavelength of 519 nm for FITC-dextran, excitation wavelength of 559 nm and emission wavelength of 567 nm for αSMA and CD206. Image scale bars were applied using ImageJ while keeping the image integrity intact.

### 2.4. Data Analysis

#### 2.4.1. Pseudo-ADC (ADC*) Calculation from T1WI

**Figure 1:**
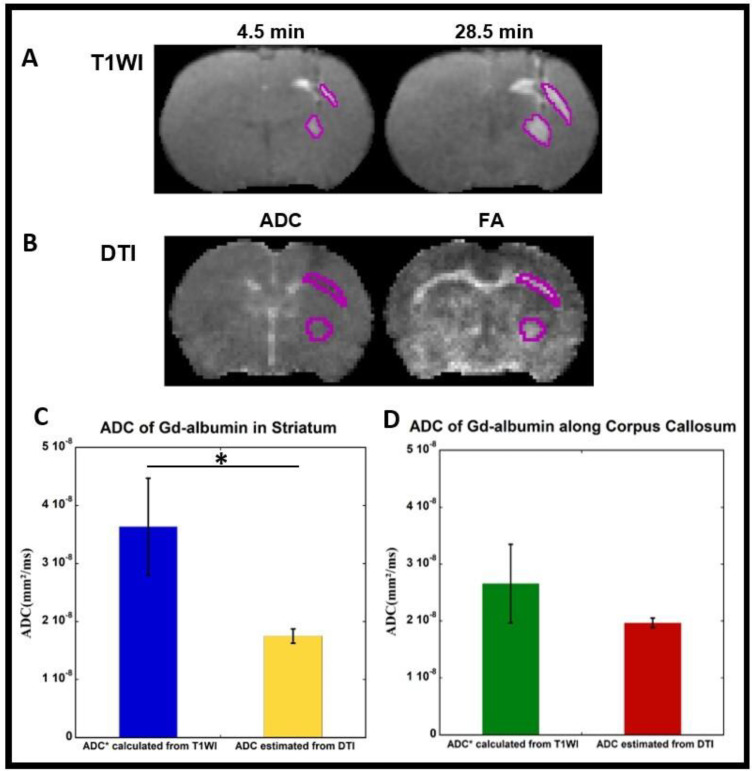
(**A**) T1-weighted images 4.5 and 28.5 min after the end of tracer infusion, shown in the coronal view, for illustration. The boundaries of the tracer were calculated using MATLAB from the initial manual segmentation. (**B**) Images of ADC, and FA maps resulted from DTI. (**C**,**D**) Bar plots showing the average ADC* of Gd-albumin calculated from T1WI and ADC estimated from DTI in the striatum (**C**) and along the corpus callosum (**D**) of rat brain after the intra-striatal administration of Gd-albumin with * *p* ˂ 0.05. Error bars indicate the standard errors.

To estimate the diffusion parameter from the contrast-enhanced T1WI, we tracked the distribution of the Gd-albumin over time. We analyzed T1WI volumes that were scanned about 4.5 and 28.5 min after the end of the tracer infusion. For each volume, a rough segmentation covering the hyperintensity regions caused by the tracer near the infusion site and along the corpus callosum was drawn manually using MRIcro software (Version: 1.40). The ROIs were then processed in MATLAB (R2022a) to identify the regional boundaries more precisely. To do this, the tissue inside the manually segmented ROIs was further segmented into two regions using Otsu’s threshold approach [89]. The region with higher intensities includes the tissue with tracer. The boundaries (surface) of the regions were identified, as shown in Figure 1A. For each T1 volume, a set of voxels, *P*(*x*,*y*,*z*), on the surface of the distributing tracers was calculated, S_1_ = {P_i_^1^|i = 1, …, N_1_}, S_2_ = {P_i_^2^|i = 1, …, N_2_}, S_3_ = {P_i_^3^|i = 1, …, N_3_}. Here, *N* is the number of voxels on each surface and since the regions are growing as the tracer distributed to the tissues, it is reasonable to assume N_1_ < N_2_ < N_3_. Next, the distance traveled by the tracer from each point on a surface to the closest point on the next surface was calculated. Knowing the dimensionality, *d* as 3, distance, *dist*, and the traveled time, *t*, by the tracer, the pseudo apparent diffusion coefficient can be estimated as:

(1) $${ADC}^{*}=\frac{Dist^{2}}{2dt}$$

The estimated diffusion values over the surface points were averaged to achieve an approximate measure of the ADC* of Gd-albumin in the striatum and along the corpus callosum.

#### 2.4.2. ADC Estimation from DTI

The DW MRI data were processed and analyzed using in-house codes in MATLAB (The MathWorks, Inc., Natick, MA, USA). First, the DW volumes were motion corrected by a coarse registration using SPM8. Then, similar to the method used by [90], the DTI tensor was estimated from the DW images. Then, DTI scalar maps such as fractional anisotropy (*FA*), apparent diffusion coefficient (*ADC*), axial (parallel) diffusion coefficient (*D_axi_*), and radial (perpendicular) diffusion coefficient (*D_rad_*) were calculated as follows:

(2) $$FA=\sqrt{\frac{1}{2}\times \frac{{\left(\lambda_{1}-\lambda_{2}\right)}^{2}+{\left(\lambda_{1}-\lambda_{3}\right)}^{2}+{\left(\lambda_{2}-\lambda_{3}\right)}^{2}}{\lambda_{1}^{2}+\lambda_{2}^{2}+\lambda_{3}^{2}}}$$

(3) $$ADC=\frac{\lambda_{1}+\lambda_{2}+\lambda_{3}}{3}$$

(4) $$D_{axi}=\lambda_{1}$$

(5) $$D_{rad}=\frac{\lambda_{2}+\lambda_{3}}{2}$$

in which *λ*_1_ > *λ*_2_ > *λ*_3_ are the sorted eigenvalues of the diffusion tensor in each voxel. FA is a measure of restricted diffusion, and it is high in white matter fiber bundles. We used ADC and FA maps to manually segment the infusion site and corpus callosum, respectively, as shown in Figure 1B.

Based on the structure of extracellular spaces, the macroscopic diffusion in the brain tissues is hindered and defined by ADC. ADC is related to the free diffusion coefficient by a restriction parameter called tortuosity, *λ*.

(6) $$\lambda^{2}=\frac{D}{D_{ADC}}$$

Our ADC maps provided the ADC values for water molecules. Using the reported free diffusion coefficient of water (18 D) as 3 × 10^−6^ mm^2^/ms [91] and Equation (6), we first calculated tortuosity for water molecules in the striatum and along the corpus callosum. Then, assuming the tortuosity for Gd-albumin to be the same as water molecules and using the reported value 8.29 × 10^−8^ mm^2^/ms [29] for the free diffusion coefficient of bovine serum albumin (66 kD) as a substitute for Gd-albumin (70 kD) and Equation (6) again, we finally estimated the ADC for Gd-albumin in the striatum and along the corpus callosum. 

The assumption of using the tortuosity for Gd-albumin to be the same as water molecules despite a huge difference in their molecular weight is reasonable since the ADC in Equation (6) is inversely proportional to tortuosity. Using true (bigger) tortuosity values for Gd-albumin would give an even smaller ADC value estimated from DTI, which would even support more in favor of our results that convective bulk flow is present in addition to diffusion in the brain.

### 2.5. Data Quantification and Statistical Analysis

To evaluate the convective bulk flow in the interstitial spaces of the brain parenchyma, ROIs were generated near the intra-striatal infusion site and the corpus callosum. ADC* of Gd-albumin was calculated from T1WIs using the dynamics of contrast agent within those ROIs. Similarly, ROIs were used to estimate the ADC of Gd-albumin from DTI-generated ADC maps. Results of calculated ADC* (from T1WIs) and estimated ADC (from DTI) using both ROIs are shown as mean ± standard error in bar plots as shown in Figure 1C,D. A two-sample *t*-test was performed between two groups with a *p* < 0.05 statistical significance level in order to determine the convective bulk flow within the brain parenchyma.

## 3. Results

### 3.1. Convective Bulk Flow in the Interstitial Spaces of the Brain Parenchyma

Gd-albumin (70 kD) was employed as a high molecular weight (MW) tracer to determine the size-independent convective bulk flow transport within the brain parenchyma. Due to the very small size of perivascular spaces, convective bulk flow cannot be directly evaluated using MRI, so we indirectly calculated ADC* values of Gd-albumin from in-vivo T1WIs. To estimate the diffusion parameter from the contrast-enhanced T1WI, we analyzed the distribution of the Gd-albumin over time. Figure 1A shows the ROIs enclosing the boundary of Gd-albumin movement near the intra-striatal infusion site and along the corpus callosum 4.5 and 28.5 min after the end of infusion, respectively. As seen in these images, the boundaries kept on expanding, indicating the movement of Gd-albumin with time. ADC* values calculated from T1WIs using the dynamics of the contrast agent were affected by both the convective bulk flow and diffusion.

ADC of Gd-albumin was estimated from DTI. The ADC and FA maps derived from DTI and the ROIs near the infusion site and corpus callosum are shown in Figure 1B. The FA maps were used to draw the ROIs near the infusion site and the corpus callosum, since these maps can show the restricted diffusion clearly, especially along the corpus callosum. Figure 1C,D show the quantitative comparison of the calculated ADC* (from T1WIs) and estimated ADC (from DTI) values (Mean ± SE) and demonstrates the significantly higher value of calculated ADC* (Figure 1C, (3.64 ± 0.8) × 10^−8^ vs. (1.75 ± 0.1) × 10^−8^, *p* = 0.038, % difference- 70) in the striatum and higher value of calculated ADC* (Figure 1D, (2.67 ± 0.7) × 10^−8^ vs. (1.97 ± 0.08) × 10^−8^, *p* = 0.34, % difference- 30) along the corpus callosum compared to the estimated value of ADC from DTI. Our data demonstrates that calculated ADC* values from T1WIs are higher than estimated ADC values from DTI, indicating that tracer movement within the brain parenchyma, particularly in the striatum and the corpus callosum, is supported not only by diffusion but also involves bulk flow independent of molecular weight of solutes.

### 3.2. Glymphatic System-Mediated Transport within the Brain Parenchyma

**Figure 2:**
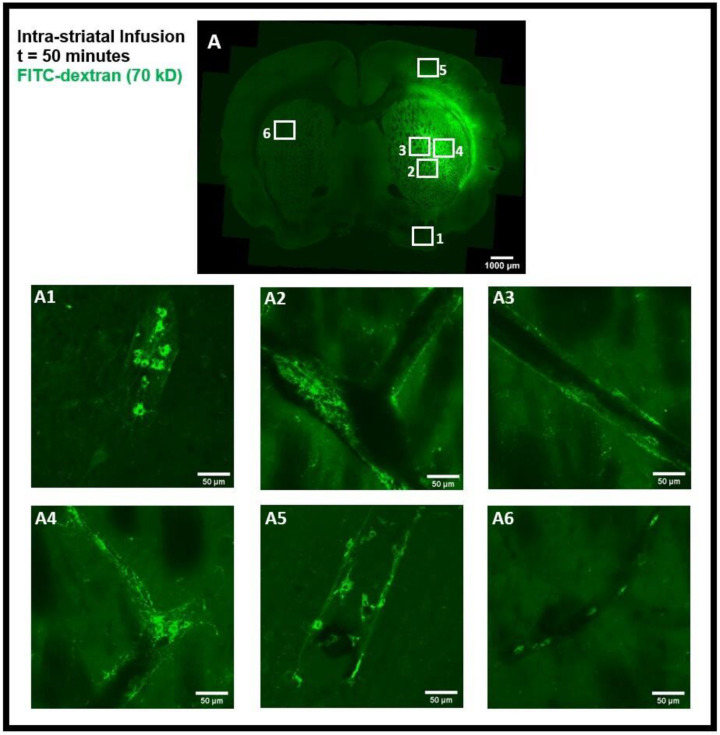
Intra-striatal administration of FITC-dextran (70 kD) in the rat brain. (**A**) shows the movement of the tracer in the striatum and the corpus callosum 50 min after infusion in coronal brain section. The presence of a tracer (green) in the perivascular spaces near the infusion site (**A2**–**A4**), far from the infusion site (**A1**,**A5**), and on the contralateral hemisphere (**A6**), can be seen using confocal microscopy imaging.

The tracer movement in the rat brain was examined using confocal microscopy imaging on coronal brain sections after intra-striatal and intra-cisternal administration of FITC-dextran, separately. The tracer movement in the striatum and the corpus callosum was detected 50 min after intra-striatal infusion of high MW FITC-dextran (70 kD), as shown in Figure 2A. The tracer was observed in the perivascular spaces near the infusion site (Figure 2A2–A4), distal from the infusion site (Figure 2A1,A5), and in the perivascular spaces of the contralateral hemisphere of the brain (Figure 2A6). 

Using an α smooth muscle cell actin (αSMA) as a marker to determine arterial vessels, we found that the tracer entered both the periarterial and perivenous spaces. Our results demonstrate that 50 min after intra-striatal infusion, the tracer not only moved far from the infusion site but also entered the perivascular spaces of both the veins and the arteries, indicating the important role of the glymphatic system and convective bulk flow in the transport of tracer/interstitial waste solutes within the brain parenchyma. To minimize the effect of intra-striatal injection on the glymphatic system [9], we also investigated the tracer distribution via the glymphatic system following intra-cisternal administration of FITC-dextran. The tracer was seen in both the hemispheres and was present mostly in the cortical periarterial and perivenous spaces 2 h post intra-cisternal infusion, demonstrating the functioning of the glymphatic system and convective bulk flow in CSF tracer transport, as was previously reported in glymphatic system studies [1,2].

### 3.3. PVMs and Their Association with the Glymphatic System

**Figure 3:**
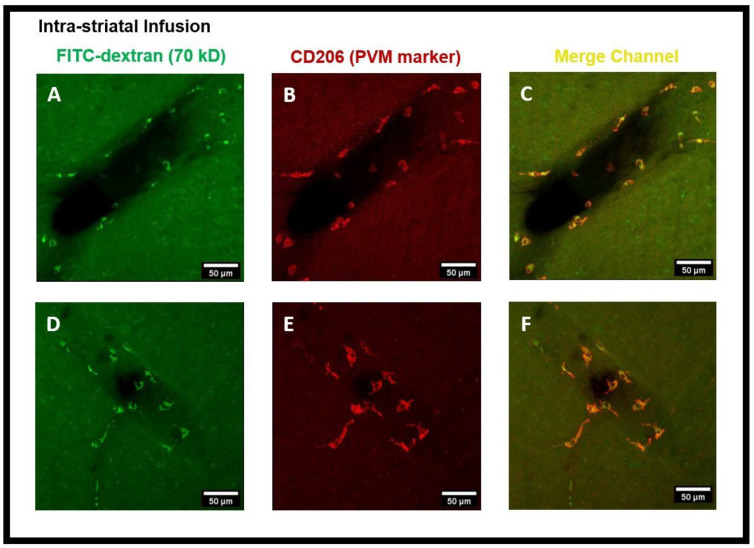
FITC-dextran tracer uptake by the perivascular macrophages (PVMs) present in the perivascular spaces after intra-striatal administration. (**A**,**D**) show the tracer (green) accumulated cells using the FITC channel, (**B**,**E**) show the CD206 staining (red) which is a specific marker for PVM using the cy3 channel, (**C**,**F**) are the images from the merged channel showing the co-localization (yellow) of CD206 antibody with tracer accumulated cells, confirming them to be PVM.

PVMs are a distinct population of resident brain macrophages localized in close proximity to cerebral blood vessels and express the mannose receptor CD206. We hypothesized that PVMs, due to their specific location in the CSF-filled perivascular spaces, may play an important role in interstitial waster clearance. Using confocal imaging analysis, we found that after intra-striatal infusion of FITC-dextran, the tracer was accumulated in cells located within the perivascular spaces of the vasculature (Figure 2). Figure 3A–F are the confocal images further showing the tracer uptake predominantly by cells within the perivascular spaces surrounding the medium to large-sized vessels (25–50 µm in diameter) and the co-localization of CD206 immunoreactive cells, a specific marker for PVMs, indicating that the tracer is primarily taken up by PVMs. 

Interestingly, after the intra-striatal infusion of FITC-dextran in the right hemisphere, the tracer was accumulated in PVMs on the contralateral hemisphere of the brain as well (Figure 2A6). This tracer accumulation by brain-wide PVMs suggests that PVMs play an essential role in sampling the components of the CSF and the removal of interstitial waste solutes from the brain.

**Figure 4:**
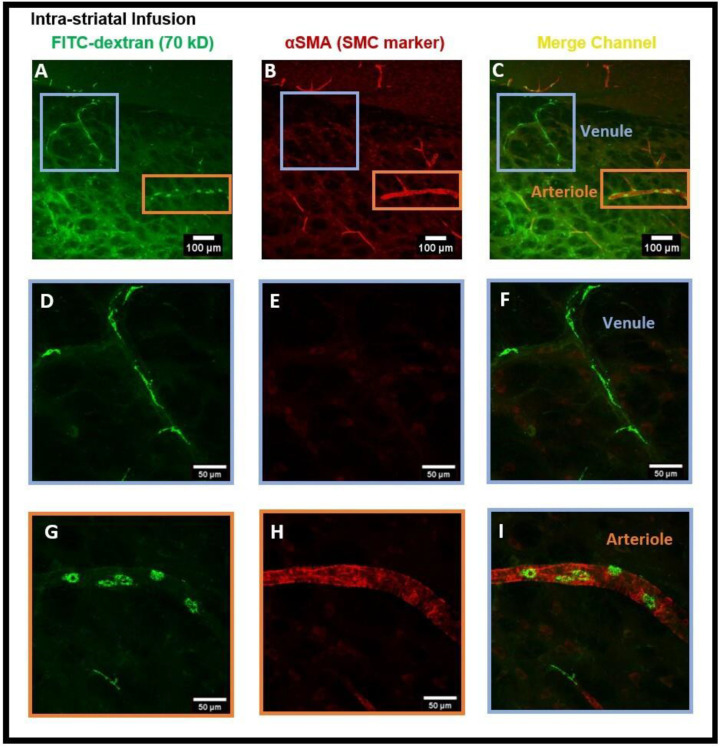
Presence of FITC-dextran and PVMs in the peri-arteriole and peri-venule spaces after intra-striatal administration of FITC-dextran. (**A**) shows the tracer and tracer accumulated cells (green) in the perivascular spaces of vasculature, (**B**) shows the αSMA staining (red) specific for smooth muscle cells (SMCs), (**C**) merged channel shows the presence of αSMA for SMC in arteriole and absence of αSMA in venules. (**D**,**G**) are the sub-images of (**A**) showing the magnified view of vessels, (**E**,**H**) are the sub-images of (**B**) showing the magnified view of αSMA staining, (**F**,**I**) are the sub-images of (**C**) showing the magnified view of αSMA^+^ arteriole (**I**) and αSMA^−^ venule (**F**). (**F**,**I**) distinguished the arterioles from veins and confirm the presence of tracer accumulated PVM in the peri-venule and peri-arteriole spaces.

Next, we administered intra-striatal tracer infusion, to determine if tracer accumulated PVMs were located in the perivascular spaces of both the arterioles and venules in which αSMA immuno positive and negative vessels were used to determine arterioles and venules, respectively. We detected CD206 positive PVMs localized to the perivascular spaces of both αSMA^+^ arterioles and αSMA^−^ venules (Figure 4A–I). 

**Figure 5:**
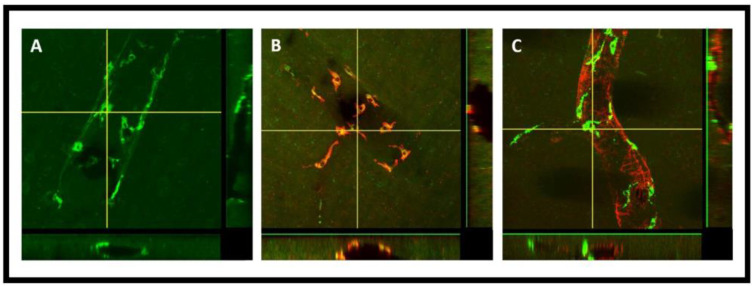
3D cross sections of XZ and YZ planes obtained using z-stacked confocal microscopy imaging. The horizontal and vertical crosslines shown in the projection image represent the XZ and YZ planes shown at the bottom and right sides, respectively. (**A**) shows the presence of tracer and tracer accumulated PVM (green) external to the lumen, preferably in the perivascular space of the vasculature, (**B**) shows the co-localization (yellow) of PVM with the CD206 antibody (red), (**C**) shows the PVM external to the SMCs (red) in the perivascular spaces utilized by the glymphatic system.

This result suggests that the PVMs filter CSF components during the glymphatic influx along arteries and glymphatic efflux along veins. Three-dimensional (3D) XZ and YZ cross-sectional confocal imaging showed that PVMs were present outside the vascular lumen (Figure 5A), and were co-localized with CD206 (Figure 5B). We detected the tracer accumulated PVMs specifically in the pial-glial periarterial spaces of the glymphatic system instead of the periarterial spaces along the basement membranes between smooth muscle cells (SMCs) in the walls of the arteries for intramural periarterial drainage pathway (IPAD) (Figure 5C). Figure 5C shows the 3D cross-sections of XZ (bottom) and YZ (right) planes clearly exhibiting the PVMs external to the SMCs in arterioles. This evidence is critical in confirming that the PVMs and the glymphatic system share the same perivascular spaces and work hand in hand for the removal of interstitial waste solutes from within the brain parenchyma. 

**Figure 6:**
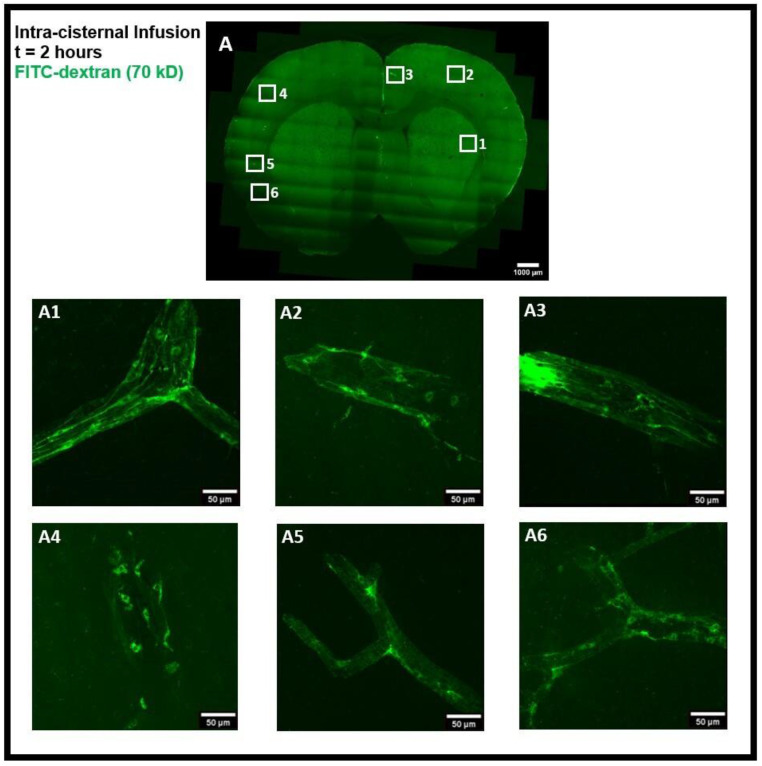
Intra-cisternal administration of FITC-dextran in the rat brain. (**A**) coronal brain section image 2 h after infusion. (**A1**–**A6**) sub-images of (**A**) showing the magnified view of the presence of tracer and tracer accumulated cells (green) in the perivascular spaces in both the hemispheres of the brain.

Again, to minimize the effect of intra-striatal injection on the glymphatic system [9], we repeated the experiments using the intra-cisternal infusion of FITC-dextran and further analyzed the tracer uptake by PVMs and their association with the glymphatic system. Figure 6A is the coronal brain section image showing tracer transport by the glymphatic system. Again, the tracer and tracer accumulated cells were prevalent in the perivascular spaces of both the cerebral hemispheres (Figure 6A1–A6).

**Figure 7:**
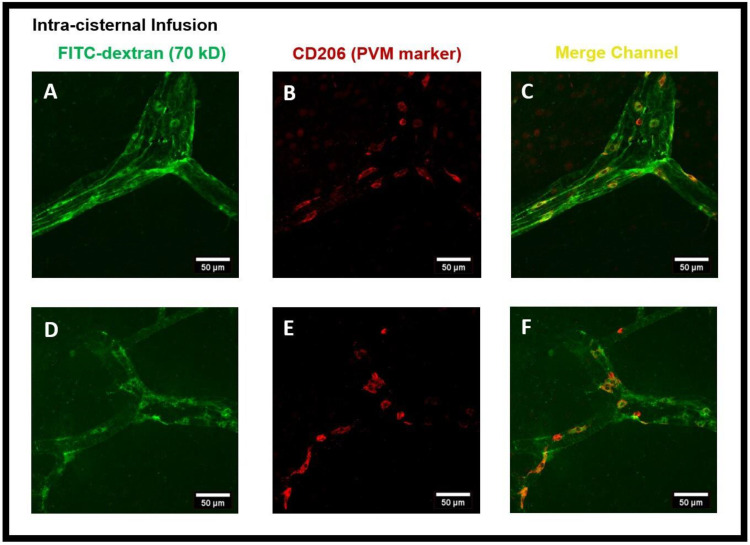
FITC-dextran tracer uptake by the perivascular macrophages (PVMs) present in the perivascular spaces after intra-cisternal administration of FITC-dextran. (**A**,**D**) show the tracer uptake by cells (green) in the perivascular spaces using the FITC channel of confocal imaging, (**B**,**E**) show the CD206 staining (red) which is a specific marker for PVM using the cy3 channel, (**C**,**F**) are the images from the merged channel (yellow) showing the co-localization of PVM with CD206 staining, confirming the tracer uptake by PVM.

To further confirm the tracer accumulated cells to be PVMs, we used CD206 staining. Figure 7A–F show the cells present in perivascular spaces and confirm them to be CD206^+^ PVMs.

**Figure 8:**
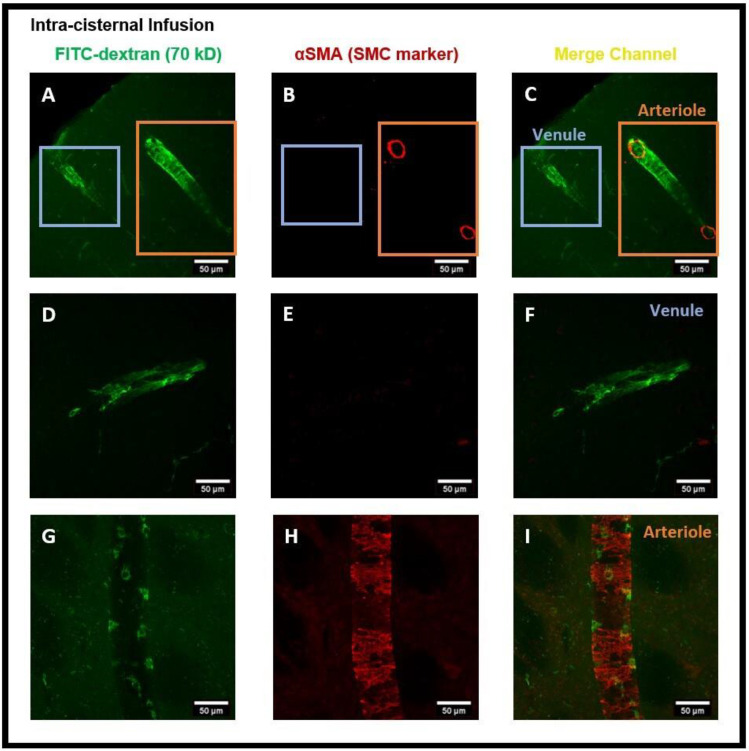
Presence of FITC-dextran and PVMs in the peri-arteriole and peri-venule spaces after intra-cisternal administration of FITC-dextran. (**A**) shows the tracer and tracer accumulated PVM (green) in the perivascular spaces of vessels, (**B**) shows the αSMA staining (red), (**C**) merged channel showing the αSMA^+^ penetrating arteriole and αSMA^−^ venule. (**D**,**G**) independent images of venule and arteriole, (**E**,**H**) αSMA staining, (**F**,**I**) merged channels identifying the tracer accumulated PVMs in the perivascular spaces of arterioles (**I**) and venules (**F**).

αSMA staining was then conducted to establish that PVMs in both peri-arterioles and peri-venules had taken up the tracer. Figure 8A–I show the tracer and tracer accumulated PVMs in perivascular spaces of the vasculature and distinguished the αSMA^+^ arterioles from αSMA^−^ veins. Altogether, confocal microscopy images after the intra-striatal and intra-cisternal infusions of tracers, separately, in the rat brain indicate the crucial function of PVMs in the uptake of tracer/interstitial waste solutes during glymphatic CSF influx (along arteries) and efflux (along veins).

## 4. Discussion

The glymphatic system proposes a brain-wide waste clearance through a specialized network of perivascular spaces and convective bulk flow of CSF, ISF, and interstitial waste solutes through the interstitial spaces of the brain parenchyma. Recent reviews provide comprehensive information on the glymphatic system, including fluid dynamics, research conducted since 2012, and current understanding [92,93]. However, the glymphatic system hypothesis of convective bulk flow in the interstitial spaces is not widely accepted and many studies argue in favor of diffusion as a microscopic mechanism of waste transport in the brain interstitial spaces [24,25,26]. Moreover, the association of the PVMs (a specific type of immune cells present in the perivascular spaces) with the glymphatic system for interstitial waste clearance has not been thoroughly investigated.

In this study, to evaluate the transport mechanism in the striatum, we utilized a high-molecular-weight tracer (Gd-albumin, 70 kD) that is ideally suited to analyze the size-independent convective bulk flow. The ADC* value calculated from T1WI accounted for the transport of the contrast agent via both convective bulk flow and diffusion processes. The ADC value estimated from DTI solely accounted for the diffusion of the contrast agent since we used high b-value of 900 s/mm^2^ with a TE of 40 ms in the DW sequence that predominantly measures the diffusion and the contribution of convective flow to the MRI signal was negligible. In the striatum, the ADC* calculated from T1WI was 70% larger than the ADC estimated from DTI as shown in the bar plots of Figure 1C. In the corpus callosum, the ADC* calculated from T1WI was 30% larger than the ADC estimated from DTI as shown in the bar plots of Figure 1D. These results demonstrate the presence of both the convective bulk flow and diffusion in the interstitial spaces of the brain, consistent with the initially proposed glymphatic system [1]. These findings are also consistent with a recent study that used a model based on the Lagrangian approach for optimal mass transport and found that convective bulk flow is the predominant mode of transport in perivascular spaces, while both convective bulk flow and diffusion are significant modes of transport within the brain parenchyma [94]. Using a mathematical model, different research [95] verified the existence of convective bulk flow and diffusion in the cortex of the human brain. However, both studies utilized small MW tracers, such as Gd-DOTA (MW-557.6 D) and Gadobutrol (MW-604 D), respectively, to establish their findings, which are not suited for studying the convective bulk flow in the brain tissue. Therefore, we attempted to address this controversy by employing Gd-albumin (70 kD), a high MW tracer that is suitable for this purpose. Another approach to effectively demonstrate the involvement of both convective bulk flow and diffusion for solute transport within the brain parenchyma would have been to include both control rats with small MW Gd-based tracer injections and rats with high MW tracer injections for their comparative analysis of convective and diffusion flow. 

In this study, the DTI-estimated ADC values in the corpus callosum (Figure 1D) are bigger than ADC values in the striatum (Figure 1C), which is expected given that the corpus callosum is composed of white matter containing millions of myelinated axons and diffusion of solutes is faster along the direction of the fiber tracts. On the other hand, the T1WI-calculated ADC* values in the corpus callosum (Figure 1D), are lower than the ADC* values in the striatum (Figure 1C), which may be attributed to the larger bulk flow near the intra-striatal infusion site due to its proximity to the needle track and the presence of more vasculature and perivascular spaces in the striatum compared to the corpus callosum.

In order to assess the role of the glymphatic system and convective bulk flow, we also employed ex-vivo confocal microscopy imaging and determined if the intra-striatal infused tracer (FITC-dextran, 70 kD) could be detected along the perivascular spaces of arteries/arterioles and veins/venules distant from the infusion site. Our findings revealed the presence of the tracer in the perivascular spaces and brain tissue distant from the intra-striatal infusion site (Figure 2) indicating the role of convective bulk flow in tracer transport within the brain parenchyma. The presence of tracer in the periarterial and perivenous spaces, demonstrating the functioning of the glymphatic system following intra-striatal infusion, was verified using αSMA labeling to differentiate between arterioles and venules (Figure 4). Although after intra-striatal infusion, we didn’t expect the tracer to enter the periarterial spaces, but this may be due to the re-circulation of the tracer via the glymphatic system after draining through the perivenous spaces into the CSF compartments (subarachnoid space or ventricles) [1]. Another plausible reason for the tracer being present in the periarterial spaces is through the glymphatic system after a little amount of tracer leakage into the subarachnoid space via the needle track during the infusion. The function of the glymphatic system was also verified after the intra-cisternal infusion of the tracer; the presence of the tracer in both hemispheres of the brain (Figure 6) and in the peri-arteriole and peri-venule spaces (Figure 8) indicate the tracer transport by the glymphatic system. 

The findings of this study using confocal microscopy imaging on coronal brain sections revealed the presence of PVMs in the perivascular spaces of arterioles external to the SMCs, the same pathway used by CSF during the glymphatic system influx. The LYVE1^+^/CD163^+^ (scavenger receptor cells) PVMs located external to the vascular SMCs along arteries and separated by basal lamina have been recently demonstrated using electron microscopy [13]. After the intra-striatal (Figure 2, Figure 3 and Figure 4) and intra-cisternal (Figure 6, Figure 7 and Figure 8) infusions, independently, we further showed the tracer uptake by the PVMs located in the perivascular spaces of both the arterioles and venules. These findings indicate that PVMs interact with CSF during both glymphatic influx (along arteries) and glymphatic efflux (along veins). In fact, PVMs not only interact with CSF but also filter its components to eliminate potentially toxic interstitial waste solutes and other foreign solutes/pathogens. This result is consistent with a recently published study in mice that found PVMs examine CSF contents as CSF moves into and out of the brain (following intra-cisternal and intra-striatal injections, respectively) and that PVMs deletion inhibit CSF influx and efflux [13]. They also observed double positive PVMs after co-injecting the tracers intra-striatally and intra-cisternally, which support the findings of our study. Following the impairment of CSF flow dynamics in PVMs-depleted mice, further analysis of the CSF protein content revealed an accumulation of synapse-related proteins (such as NRXN1, CDH2, and NRCAM, etc.) and AD-related proteins (such as clusterin, apolipoprotein, and amyloid precursor protein, etc.). Furthermore, they found LYVE1^+^ (scavenger receptor cells) PVMs along αSMA^+^ arteries/arterioles and MHCII^+^ (antigen-presenting cells) PVMs along αSMA^−^ veins/venules, showing the distinct functions of PVMs identified in perivascular spaces of arteries and veins. They also found increased fibroblast activity, decreased MMP activity, impaired arterial vasodilatory responses, and an accumulation of extracellular matrix (ECM) proteins (such as laminin, collagen IV, etc.) along αSMA^+^ vessels in PVMs-depleted mice, indicating that LYVE1^+^ MHCII^lo/neg^ PVMs regulate the CSF flow dynamics through their capacity to regulate ECM remodeling, which in turn affects the arterial stiffness and pulsation [13]. A very recent study has shown that dynamic variations in vascular diameter/vasomotion due to neurovascular coupling encourage CSF to flow through the perivascular spaces, hence driving perivascular glymphatic CSF influx and clearance [14]. Collectively, these findings imply that PVMs not only interact with CSF during glymphatic influx and efflux but also regulate CSF flow dynamics by modulating arterial stiffness/pulsations, which is the key contributor to CSF-ISF exchange in the glymphatic system. As a result, the PVMs are closely related to the glymphatic system, and the two systems collaborate to clear toxic interstitial waste solutes from the brain and maintain homeostasis.

Furthermore, PVMs have been demonstrated to be the critical players in regulating the CSF flow dynamics in aging and AD [13]. Also, with aging, the functioning of the glymphatic system decreases [60,61], probably due to a decrease in vascular elasticity and neurovascular coupling [14,96,97]. The glymphatic system and PVMs should be explored further as potential novel treatment targets for patients with AD and age-related disorders.

One drawback of this work is that the intra-striatal infusion is traumatic, severely suppresses the functioning of the glymphatic system, and disturbs fine pressure gradients. However, to minimize these effects, we carefully introduced a PE-10 catheter into the striatum to examine the convective bulk flow within the brain parenchyma. Additionally, during the intra-striatal infusion in MRI scans, a tiny amount of tracer may have leaked into the subarachnoid space via the needle track and re-entered the brain parenchyma via the glymphatic pathways which is another limitation of this study. However, we employed a low infusion rate of 0.3 μL/min to minimize this kind of tracer leakage. Another drawback of this study is that the evaluation of ADC values of Gd-albumin from T1WIs and DTI could have some flaws. First, we used a regional growing scheme to assess the distance that the Gd-albumin particles traveled between two MRI scans. In this process, the segmentation of tissues containing the tracer could not be very precise due to limited contrast to noise ratio (CNR). Second, we had to assume the tortuosity for Gd-albumin to be the same as water molecules. Although this could impose an overestimation bias for the DTI-estimated ADC values, our conclusion that diffusion alone is not sufficient for the tracer movement within the brain parenchyma is still valid, as we discussed in Section 2.4.2. Moreover, the choice of anesthesia can significantly impact measured glymphatic flow dynamics within the brain [62,98]. Although we employed only high-dose isoflurane as an anesthetic agent in our study, despite this limitation, we anticipate that differences between ADC* calculated from T1WI and ADC estimated from DTI should remain consistent across our experimental conditions. Also, while we conducted experiments only on male rats, it is important to note that a recent study exploring the glymphatic system in both female and male rodents revealed no significant biological sex differences [99]. However, further studies including both female and male rodents are warranted.

## 5. Conclusions

In conclusion, to examine the solute transport mechanism in the brain interstitial spaces, we utilized high MW Gd-albumin for intra-striatal administration. In the striatum and along the corpus callosum, the ADC* calculated from T1WI was 70% and 30% higher, respectively, than the ADC estimated from DTI, suggesting the existence of both the convective bulk flow and diffusion in the interstitial spaces of the brain. Confocal microscopy imaging revealed tracer in the peri-arteriole and peri-venule spaces, as well as brain tissue distant from the intra-striatal infusion site, indicating the contribution of the glymphatic system and convective bulk flow in parenchymal tracer transport, which was also confirmed after intra-cisternal tracer infusion. PVMs were observed in the pial-glial perivascular spaces utilized by the glymphatic CSF influx and efflux for interstitial waste clearance. This study additionally showed tracer uptake by PVMs in both the peri-arteriole and peri-venule spaces following intra-striatal and intra-cisternal administration of the tracers, separately. Collectively, the findings of this study suggest that PVMs are the important contributors to interstitial waste clearance during glymphatic CSF influx along arteries and glymphatic CSF efflux along veins. A better understanding of the association between these pathways may lead to new therapeutic strategies for numerous neurological conditions.

## Data Availability

The data presented in this study are available on request from the corresponding author.
